# Whole genome sequencing in the palm of your hand: how to implement a MinION Galaxy-based workflow in a food safety laboratory for rapid *Salmonella* spp. serotyping, virulence, and antimicrobial resistance gene identification

**DOI:** 10.3389/fmicb.2023.1254692

**Published:** 2023-12-01

**Authors:** Alexandre Lamas, Alejandro Garrido-Maestu, Alberto Prieto, Alberto Cepeda, Carlos Manuel Franco

**Affiliations:** ^1^Food Hygiene, Inspection and Control Laboratory (Lhica), Department of Analytical Chemistry, Nutrition and Bromatology, Veterinary School, Universidade da Santiago de Compostela, Lugo, Spain; ^2^Food Quality and Safety Research Group, International Iberian Nanotechnology Laboratory, Braga, Portugal; ^3^Department of Animal Pathology (INVESAGA Group), Faculty of Veterinary Sciences, Universidade de Santiago de Compostela, Lugo, Spain

**Keywords:** *Salmonella* spp., whole genome sequencing, MinION, Flongle, serotyping, antimicrobial resistance, virulence

## Abstract

**Introduction:**

Whole Genome Sequencing (WGS) implementation in food safety laboratories is a significant advancement in food pathogen control and outbreak tracking. However, the initial investment for acquiring next-generation sequencing platforms and the need for bioinformatic skills represented an obstacle for the widespread use of WGS. Long-reading technologies, such as the one developed by Oxford Nanopore Technologies, can be easily implemented with a minor initial investment and with simple protocols that can be performed with basic laboratory equipment.

**Methods:**

Herein, we report a simple MinION Galaxy-based workflow with analysis parameters that allow its implementation in food safety laboratories with limited computer resources and without previous knowledge in bioinformatics for rapid *Salmonella* serotyping, virulence, and identification of antimicrobial resistance genes. For that purpose, the single use Flongle flow cells, along with the MinION Mk1B for WGS, and the community-driven web-based analysis platform Galaxy for bioinformatic analysis was used. Three strains belonging to three different serotypes, monophasic *S*. Typhimurium, *S*. Grancanaria, and *S*. Senftenberg, were sequenced.

**Results:**

After 24 h of sequencing, enough coverage was achieved in order to perform *de novo* assembly in all three strains. After evaluating different tools, Flye *de novo* assemblies with medaka polishing were shown to be optimal for *in silico Salmonella* spp. serotyping with SISRT tool followed by antimicrobial and virulence gene identification with ABRicate.

**Discussion:**

The implementation of the present workflow in food safety laboratories with limited computer resources allows a rapid characterization of *Salmonella* spp. isolates.

## 1 Introduction

Control and detection of foodborne pathogens in food production chains is essential to ensure consumer safety. *Salmonella* spp. is considered one of the major foodborne pathogens worldwide. In the European Union (EU), this genus is the second most common cause of foodborne diseases (European Food Safety Authority and European Centre for Disease Prevention and Control, 2022). The reference method for the detection of *Salmonella* in food and feed is ISO 6579:2017 (ISO, 2017). This is a culture-based method for which at least 3 to 5 days are necessary to determine the presence of *Salmonella* spp. In addition to this, extra time and hands-on work is still needed if serotyping and/or virulence determinant characterization is required. The reference serotyping method is based on agglutination using antisera (ISO, 2014), which is a laborious method, and may take several days to reach a definitive serotype result. In addition, having the necessary antisera to determine all serotypes is only feasible for reference laboratories. However, *Salmonella* serotyping is important from an epidemiological and legislative point of view. The Regulation 2160/2003 (European Union, 2003) establishes that it is necessary to control the serotypes of *Salmonella* with major epidemiological importance in poultry production.

To overcome the limitations of culture-dependent microbiology and molecular methods like qPCR, the application of Whole-Genome Sequencing (WGS) and *de novo* assembly is attractive. WGS has a huge number of applications in microbiology, particularly in the study of foodborne pathogens (Allard et al., 2020; Lakicevic et al., 2023). Thanks to the development of this technology, it is now possible to sequence the genome of a bacterium for less than a few hundred dollars. This tool a performs a complete bacterial genomic characterization, including the determination of resistance and virulence genes and plasmids, and can be used in phylogenetic studies. This tool is especially useful for tracking clones during foodborne outbreaks (Lakicevic et al., 2023).

Next-generation sequencing (NGS) technologies can be classified into two groups: short-read sequencing and long-read sequencing. Short-read sequencing technologies, such as Ion Torrent and Illumina, spread quickly as they are more developed and produce highly accurate reads (Akaçin et al., 2022). However, they only generate reads of a few hundreds of bases. Bacterial genomes present repetitive regions, and this technology cannot disambiguate repetitive regions of the genome when they are longer than the read size (Goldstein et al., 2019). In addition, sample preparation protocols often involve multiple time-consuming steps. Long-read sequencing, meanwhile, has become popular in recent years, with PacBio and Oxford Nanopore Technologies (ONT) the most popular technologies (De Coster et al., 2021). ONT has acquired relevance because it is possible to acquire a sequencing platform at a minimal initial cost and has several available library preparation kits, like the Rapid Sequencing kit (SQK-RAD004, https://store.nanoporetech.com/eu/rapid-sequencing-kit.html), that reduce the handling time to about 10 min. Out of the different sequencing equipments that ONT has in its catalog, the MinION has become the most popular. This is due to the fact that it can be purchased for < $1,000, it is smaller than a current smart phone, and it can be controlled from a medium-capacity laptop. This makes it affordable even for small research teams. This device uses flow cells for sequencing. Regular flow cells have a minimum of 800 pores and theoretically produce up to 30 Gb. More recently, ONT launched the so-called Flongle flow cells which have a minimum of 80 pores and can produce up to 2.8 Gb, with a price of approximately $90. These are single-use flow cells but, even with a lower amount of data, they can be enough for sequencing the complete genome of a given strain with enough coverage for downstream analysis and can run single samples on demand. However, MinION read quality is lower than that of short-read sequencing technologies, and there are systematic errors in homopolymer regions. But continuous improvement of sequencing chemistry, along with base-calling software, has increased the accuracy of this technology (Senol Cali et al., 2018).

NGS always comes hand in hand with bioinformatics. In recent years, a number of tools with great applicability to foodborne pathogen research have been developed. Furthermore, there are tools to perform *de novo* assembly (construction of genomes from sequencing reads) such as Flye (Lin et al., 2016) and Canu (Koren et al., 2017) or Raven (Vaser and Šikić, 2021) that were specifically developed for long reads, or Unicycler (Wick et al., 2017) that was developed for hybrid (short and long read) assemblies. There are also tools to annotate the assemblies like Prokka (Seemann, 2014), and others to identify the presence of antimicrobial resistance genes such as Resfinder (Florensa et al., 2022) or virulence genes such as ABRicate (Seemann, 2016). In addition to this, specific tools for *Salmonella* were developed, such as SISTR (Yoshida et al., 2016) or SeqSero2 (Zhang et al., 2019) for *in silico Salmonella* serotyping. Many of these tools were developed to be used in a Linux environment and some of them, like the assemblers, are computationally demanding. This limits their use by researchers with low bioinformatic skills and by laboratories that do not have high-performance computing capabilities. To facilitate the accessibility to these tools, user-friendly, freely available, community-driven web-based analysis platform have appeared. Within them, Galaxy (The Galaxy Community, 2022), known for its high-computing power, is the most successful platform for the analysis of different -omics data (http://galaxyproject.org). The users can upload their sequencing data to the cloud environment, and perform a great variety of bioinformatic analysis through an intuitive interface. The Center for Food Safety and Applied Nutrition (CFSAN) of the U.S. Food and Drug Administration has developed a customized instance in the Galaxy environment called GalaxyTrakr (Gangiredla et al., 2021), to be implemented by laboratory scientists performing food-safety research. The aim is that laboratories outside the FDA's internal network, with no dedicated bioinformaticians or computing power, can implement these tools in their routine research and work.

The aim of the present work was to develop a *Salmonella* spp. WGS workflow that could be implemented in food safety laboratories with reduced access to NGS equipment and limited bioinformatic skills. For this purpose, MinION and Flongle technology were used in combination with the user-friendly platform Galaxy to perform the bioinformatics analyses. Three Salmonella strains of different serotypes were used to determine the most suitable tools for *de novo* assembly and to be used later in the genomic characterization of the strains.

## 2 Materials and methods

### 2.1 *Salmonella* strains

Three wild *Salmonella* strains, belonging to different O groups and with different flagellar genes, isolated in Spain, were analyzed in this study. Two strains, *S*. Grancanaria Lhica GR1 and *S*. Senftenberg Lhica S1, were isolated from poultry under *Salmonella* national control plans and one strain, monophasic *S*. Typhimurium Lhica MST 1, was isolated from cow feces from an outbreak in a dairy farm. The *Salmonella* were isolated following the ISO 6579-1:2017. Four sock swabs from the poultry farm and 25g of cow feces were mixed with 225 mL of Buffered Peptone Water (BPW, Merck Millipore, Darmstadt, Germany) and incubated 18h at 37°C. Then, 100 μL of the pre-enrichment was transferred to Modified Semi-Solid Rappaport-Vassiliadis (MSRV, BD, Madrid, Spain) and plates were incubated at 42°C for 48 h (plates were checked after the first 24 h of incubation). *Salmonella* presumptive colonies on MSRV were confirmed through subculturing on Xylose Lysine Deoxycholate Agar (XLD, Oxoid, Hampshire, UK) and SM-ID2 (bioMérieux, Marcy-l'Étoile, France); both media were incubated 37°C for 24 h. From these last two media, a typical colony was taken and streaked on Nutrient Agar (NA, Panreac, Barcelona, Spain) and incubated at 37°C for 24 h. API-20E (bioMérieux) and a *Salmonella* latex test (Microgen bioproducts, Nocacyt, Surrey, UK) were used for biochemical species identification following the instructions provided by the manufacturers. Serotyping of *Salmonella*-confirmed isolates was performed following the White Kauffmann-Le Minor scheme with commercial antisera. A monophasic *Salmonella* strain was confirmed by PCR in Algete Central Veterinary Laboratory (Spain). Strains were saved in cryovials at −20°C. The phenotypic antimicrobial resistance profile of monophasic *S*. Typhimurium Lhica MST 1 was determined using the Kirby–Bauer disk diffusion method according to CLSI; growth inhibition zones were interpreted following the antimicrobial breakpoints for Enterobacteriaceae by the CLSI guidelines (Clinical Laboratory Standards Institute, 2014, 2018).

### 2.2 DNA extraction

*Salmonella* strains were recovered from frozen by placing 1 cryovial in 10 mL of Brain Heart Infusion (BHI, Merck Millipore) under constant agitation at 130 rpm for 18 h at 37°C. Then, 100 μL of the growth culture were transferred to a new tube with 10 mL of BHI and incubated again under the same conditions. Finally, 2 mL of the culture was transferred to a 2 mL microtube and centrifuged at 16,000 × *g* for 2 min. The supernatant was discarded, and the pellet was used for DNA isolation with Purelink Genomic DNA Mini Kit (Invitrogen, ThermoFisher Scientific, Waltham, MA, USA) following the manufacturer's instructions for Gram-negative bacteria. The fluorimeter Qubit^TM^ 4 (Invitrogen, ThermoFisher), in combination with Qubit 1 × dsDNA HS assay kit (Invitrogen, ThermoFisher) was used for DNA quantification.

### 2.3 Whole genome sequencing

A concentration of 200 ng of DNA was used for sequencing with the Rapid Sequencing kit [SQK-RAD004, Oxford Nanopore Technologies (ONT), Oxford, UK] following the manufacturer's instructions. The equipment MinION MK1B with the Flongle adapter Flongle Pk.1 connected to a Notebook HP 15S-FQ1055NS with an Intel^®^ Core^TM^ i7 1065G7 CPU 1.3 GHz, 8 GB RAM, SSD 512 GB was used for sequencing. The software MinKNOW 22.12.7 was used to control the sequencing run. New Flongle flow cells were used for each strain, and time of sequencing was set at 24 h. Although real-time basecalling can be performed, this option was disabled when programming the run, and off-line basecalling was performed.

### 2.4 Bioinformatic analysis

#### 2.4.1 Basecalling

The ONT Guppy basecalling software for windows version 6.0.1 was used for basecalling via the windows command line. The “Fast basecalling” in CPU mode with default parameters was used. Fast5 files were the input from sequencing and Fastq files were the output. The default mode created a Fastq file every 4,000 reads. Therefore, the number of files obtained in this step varied depending on the number of reads obtained in every sequencing run.

#### 2.4.2 Assembly

Most bioinformatic analyses were carried out on the community-driven web-based analysis platform Galaxy (The Galaxy Community, 2022) through the European server (https://usegalaxy.eu/). Fastq files were upload to the Galaxy server, and a different history was created for each bacterium. Fastq files were transformed to Fasta using the tool FASTQ to FASTA converter (Galaxy Version 1.1.5). The tool Merge.files Merge data (Galaxy Version 1.39.5.0) was used to merge all the Fasta files in a unique file. Then, the tool Porechop adapter trimmer for Oxford Nanopore reads (Galaxy Version 0.2.4+galaxy0) was used to trim the reads of merged file using default parameters, in order to remove the adapters from Nanopore on the ends of the reads (when the adapters were in the middle of a read they were treated as a chimeric and chopped into separate reads). Then NanoPlot Plotting suite for Oxford Nanopore sequencing data and alignments (Galaxy Version 1.36.2+galaxy1) was used to create a statistical summary of the sequencing reads.

*De novo assembly* was performed in four different assemblers specifically designed for long reads or for a hybrid combination with short reads. Flye (Lin et al., 2016) *de novo* assembler for single molecule sequencing reads (Galaxy Version 2.9.1+galaxy0), Canu (Koren et al., 2017) assembler optimized for long error-prone reads such as PacBio, and Oxford Nanopore (Galaxy Version 2.1.1+galaxy0) and Raven (Vaser and Šikić, 2021) *de novo* assembly of Oxford Nanopore Technologies data (Galaxy Version 1.8.0+galaxy0) were used for default parameters. The fourth software to create assemblies was Unicycler (Wick et al., 2017), a pipeline for bacterial genomes (Galaxy Version 0.5.0+galaxy1). This is a hybrid assembler that combines long and short reads, however, it can also be used only with long reads like in this study. The tool Bandage (Wick et al., 2015) visualizes *de novo* assembly graphs (Galaxy Version 2022.09+galaxy4) and was used to visualize the assembly of all the assemblers except Canu, where there is not an output assembly graph. All the tools were used with default parameters.

#### 2.4.3 Polishing

Medaka (Ltd., ONT, 2020) consensus pipeline Assembly polishing via neural networks (Galaxy Version 1.7.2+galaxy0) was used to polish the final assembly obtained with each assembler. Two independent rounds were applied. Original and polished assemblies were analyzed with BUSCO (Simão et al., 2015) to assess genome assembly, and annotation completeness (Galaxy Version 5.4.4+galaxy0) and Prokka (Seemann, 2014). Prokaryotic genome annotation (Galaxy Version 1.14.6+galaxy1).

#### 2.4.4 Serotyping, antimicrobial resistance, and virulence

The tool sistr_cmd *Salmonella In silico* Typing Resource commandline tool for serovar prediction (Galaxy Version 1.1.1+galaxy1) (Yoshida et al., 2016) was used to *in silico* to predict the *Salmonella* serotype. The tool ABRicate (Seemann, 2016) Mass screening of contigs for antimicrobial and virulence genes (Galaxy Version 1.0.1) was used to determine the presence of resistance and virulence genes. The option ResFinder database was selected to search for resistance genes and VFDB was used to search for virulence genes. Outside the Galaxy environment, other user-friendly tools were used. The web-based tool PLSDB—A plasmid database (Galata et al., 2019), available at https://ccb-microbe.cs.uni-saarland.de/plsdb/, was used for plasmid searches in all the assemblies. The tool for *in silico* serotyping, SeqSero2 (Zhang et al., 2019), which is available at http://denglab.info/SeqSero2, was used to serotype the sequenced strains. Both fastq files with raw read sequences and assemblies were used to evaluate the tool.

## 3 Results and discussion

### 3.1 Flongle sequencing statistics

Long-reading sequencing has revolutionized bacterial genome sequencing and *de novo* assembly. Specifically, the MinION equipment developed by ONT has allowed the democratization of mass sequencing and all types of laboratories can now have access to this technology with a minimum investment. In addition, in parallel to this equipment, rapid kits like the Rapid Sequencing kit have been developed for the preparation of libraries for sequencing. This kit is designed to be used with basic laboratory equipment and eliminates complex steps as well as the need for PCR target amplification. In the present study, three strains were successfully sequenced using Flongle flow cells. In Table 1 the main sequencing statistics are summarized. The sequencing time was 24 h for flow cells ALM126 and ALM338 and 21 h 42 min for flow cell ALM536. The latter was stopped because the number of pores that continued to sequence was very low. Before sequencing, the number of active pores was 89, 88, and 95 in Flongle flow cells ALM536, ALM126, and ALM338 respectively (values obtained during the flow cell QC performed before sequencing), thus all were above the manufacturer's warranty of a minimum 80 pores. Despite using the same concentration of starting DNA, and the flow cells having a similar number of pores, there were differences in the number of bases sequenced. While ALM338 and ALM 126 sequenced more than 400 Mb, ALM536 only sequenced 200 Mb bases. The number of reads was almost double in ALM338 in comparison to ALM126. This was a remarkable observation; despite the same amount of initial DNA and a similar number of pores, the final yields were quite different. The median read length was over 3,000 bases in the three cases but the n50 obtained with *Salmonella* Grancanaria was higher than in the other strains, with a value of 15,057. Based on the assembly data shown in Table 2, the coverage ranged between 98 × of monophasic *S*. Typhimurium and 50 × of *S*. Senftenberg. Wu et al. (2023) found that a minimum of 30 × coverage in *Salmonella* Nanopore sequencing offered optimal results for serotype prediction and determination of ARM/virulence gene profiles. Therefore, the coverages obtained in the current study with Flongle flow cells were enough for downstream analysis.

**Table 1:** Sequencing statistics generated with Nanoplot in usegalaxy.eu with tool Nanoplot.

	**Monophasic *Salmonella* Typhimurium LHICA MST1**	***Salmonella* Grancanaria LHICA GR1**	***Salmonella* Senftenberg LHICA S1**
Flow cell ID	ALM338	ALM126	ALM536
Time	24 h	24 h	21 h 42 min
No. of Reads	81,704	57,305	43,234
No. of bases (Mbps)	491	412	243
Median read Length (bps)	3,519	3,767	3,154
Mean Read Length (bps)	6,014	7,197	5,638
Read length stdev (bps)	7,053	8,967	7,522
n50 (bps)	11,616	15,057	12,180
Longest read (bps)	148,249	110,920	128,463
Coverage (x)^*^	98	84	50

^*^With the exception of coverage that was calculated based in the total Flye assembly size.

**Table 2:** Assembly statistic obtained with the four assemblers evaluated.

	**Flye**		**Unicycler**		**Canu**		**Raven**	
	**Original**	**Medaka**	**Original**	**Medaka**	**Original**	**Medaka**	**Original**	**Medaka**
***S*** **Grancanaria LHICA GR1**								
**Assembly time (min)**	19		28		65		57	
**Number of contigs**	2	2	2	2	3	3	2	2
**Largest contig**	4,885,218	4,891,007	4,886,925	4,891,225	4,926,151	4,937,932	4,886,644	4,891,322
**Total length**	4,971,198	4,977,087	4,972,920	4,977,324	5,115,049	5,127,639	4,972,615	4,977,420
**GC (%)**	52.15	52.14	52.14	52.14	52.19	52.18	52.14	52.14
**Monophasic** ***S***. **Typhimurium LHICA MST1**								
**Assembly time (min)**	14		42		58		55	
**Number of contigs**	4	4	108	108	2	2	2	2
**Largest contig**	5,002,478	5,008,398	5,004,292	5,008,588	5,017,395	5,027,804	5,029,613	5,034,116
**Total length**	5,072,222	5,078,268	5,557,176	5,562,015	5,027,936	5,038,391	5,034,886	5,039,428
**GC (%)**	52.16	52.14	52.05	52.06	52.17	52.15	52.16	52.15
***S***. **Senftenberg LHICA S1**								
**Assembly time (min)**	7		6		81		26	
**Number of contigs**	3	3	1	1	5	5	1	1
**Largest contig**	4,785,761	4,791,000	4,786,224	4,791,199	4,797,874	4,809,859	4,785,884	4,791,165
**Total length**	4,797,530	4,802,817	4,786,224	4,791,199	4,828,298	4,840,554	4,785,884	4,791,165
**GC (%)**	52.12	52.10	52.10	52.10	52.09	52.09	52.10	52.10

The assembly time refers to wall clock time not CPU time.

The longest read that passed the quality control during basecalling was higher than 100,000 bases in the three strains, with the longest read being 148,249, obtained in monophasic *Salmonella* Typhimurium. One of the most important points for long-read sequencing is the isolation of High molecular weight (HWM) DNA (Schalamun et al., 2019). This is key to optimizing sequencing efficiency, especially when using the Rapid Sequencing kit, or its multiplex version Rapid Barcoding kit. These kits are based in the use of transposases that randomly fragment the DNA during library preparation. If a highly fragmented DNA sample is used, during library preparation it is further fragmented, rendering sequencing less efficient and subsequent bioinformatic analysis more challenging. In recent years, commercial DNA extraction kits that physically disrupt the cell through mechanical lysis have emerged. This strategy increases the recovery of DNA but can generate highly fragmented DNA molecules. There are kits commercially available specially developed for HMW DNA isolation that can be selected for this application. This is an important factor that researchers have to evaluate when choosing a DNA kit or an in-house protocol. In the present work, a general DNA isolation kit, Pure Link Genomic DNA mini kit, based on chemical lysis, was used, obtaining optimal results during sequencing.

### 3.2 *De novo* assembly and polishing

By using long-read sequencing, in combination with specifically designed *de novo* assemblers, it is possible to obtain one single contig representing the bacterial chromosome (Moss et al., 2020). By using short-read sequencing, like Illumina, a high number of contigs are typically obtained (Judge et al., 2016), making it virtually unfeasible to obtain complete and closed bacterial genomes. To combine the potential of both technologies, hybrid assemblers such as Unicycler (Wick et al., 2017) were developed. By combining both types of reads it is possible to obtain a complete error-free genome (Jurkiw et al., 2022). However, using both technologies increases the cost of WGS. For specific applications, such as the workflow for a rapid characterization of *Salmonella* strains described herein, long reads can be enough for *in silico* serotyping and antimicrobial resistance gene detection.

**Figure 1:**
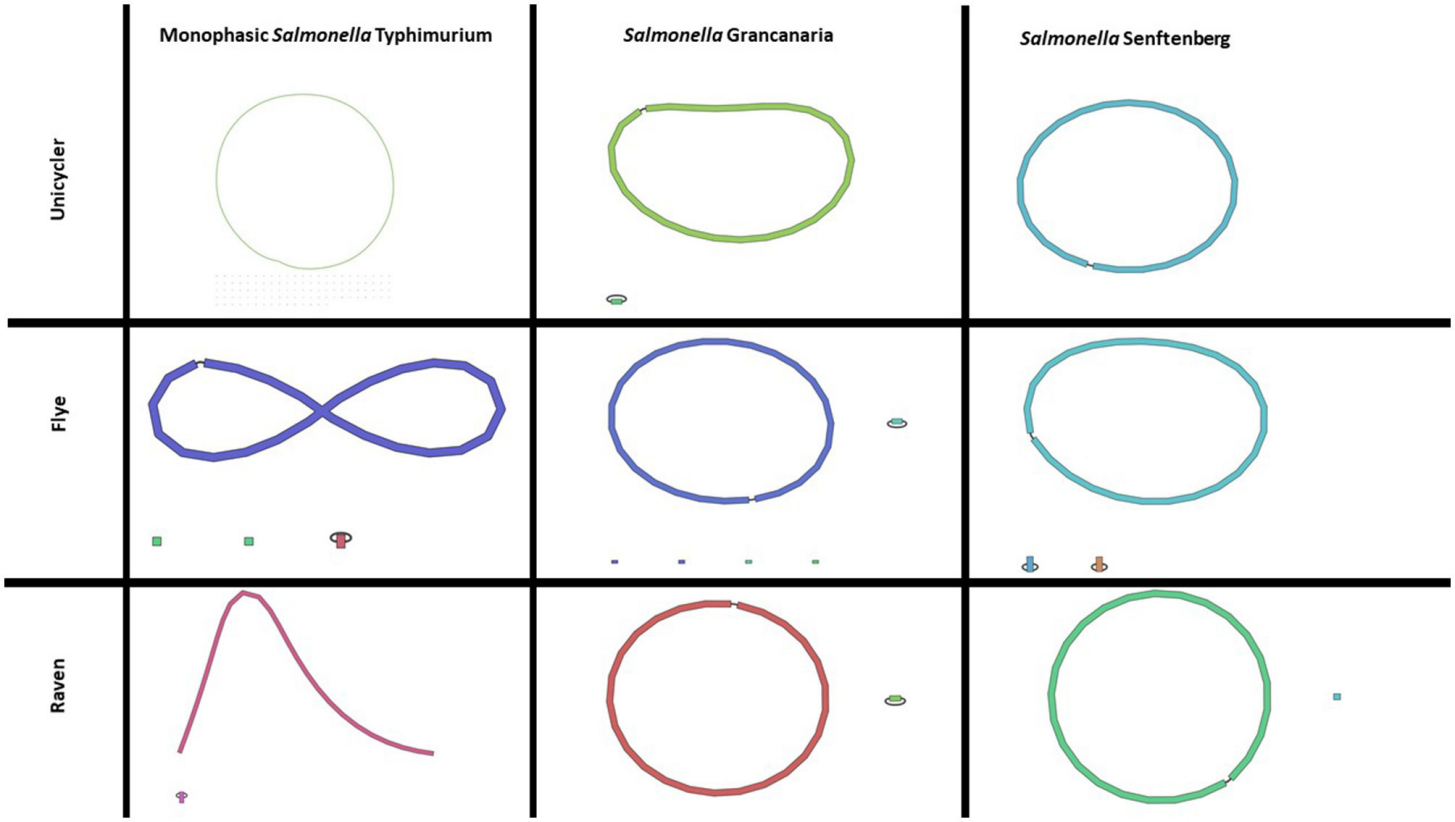
Bandage images of assemblies obtained with Unicycler, Flye, and Raven in any of the three *Salmonella* strains included in the present study. Canu assembly is not included as there is no graphical fragment assembly output in this tool with Canu in usegalaxy.

In this work, four different assemblers were compared to perform the *de novo* assembly of *Salmonella*: Flye (Lin et al., 2016), Canu (Koren et al., 2017), Unicycler (Wick et al., 2017), and Raven (Vaser and Šikić, 2021). The first point to analyze was the time needed to get the assembly. There were differences among the different strains, but Canu was the slowest of the four assemblers, needing around 1 h (see Table 2), while Flye showed the best performance. In the case of *S*. Senftenberg, only 7 min were necessary to obtain the final assembly. There were also differences between them in the number and size of the longest contigs obtained (Table 2). Bandage image was used to visualize *de novo* assembly graphs (Figure 1), except in the case of Canu assemblies because in Galaxy there is no graphical fragment assembly output for this assembler. This tool interprets the assembly and displays the differences between the different assemblers used. Focusing on monophasic *S*. Typhimurium, four contigs were obtained with Flye, two with Canu and Raven, and 108 with Unicycler. The largest contig was approximately 5 MB with all the assemblers. Flye assembly bandage image (Figure 1) shows an eight form of the largest contig, probably due to the presence of a repetitive element in the genome that the assembler did not interpret properly. Also in the case of Flye, there is one contig of 34,789 bases and another of 24,376 bases that carry genes as *noh*A, *rrr*D, tRNA, or *dna*C, which are involved in bacterial chromosome replication. Therefore, these contigs would be part of the bacterial chromosome. Smaller contigs with a high similarity to a plasmid isolated from *S*. Typhimurium (LR792482.1) were identified in all the assemblers. That plasmid carries the resistance gene aminoglycoside O-phosphotransferase APH(3′)-Ia. Four assemblers showed differences in the plasmid assembly. In the case of Canu and Flye, the contigs were longer than the reference plasmid LR792482.1 and both presented duplication of some of the genes included in the plasmid, such as APH(3′)-Ia, *rpo*, and *mbe*C. In the case of Unicycler, as can be observed in Figure 1, there are 107 contigs with a length of around 5,000 bases. These contigs are related to the plasmids previously described and, therefore, the high number of contigs was due to the inability of the assembler to create a single plasmid consensus contig. Only with Raven was a contig with a similar length to LR792482.1.obtained With respect to Raven, it is also important to note that a circular contig is not observed in Bandage image.

Regarding *S*. Grancanaria, two contigs were obtained with Flye, Unicycler, and Raven and three contigs were obtained with Canu. In contrast to monophasic *S*. Typhimurium, a larger contig circularization can be observed in the three Bandage images (Figure 1). With four assemblers the largest contig is around 4.9 Mb (Table 2). The second largest contig had a similar length with all the assemblers except for Canu, which is 30,000 bases larger than the other assemblers. These second largest contigs were analyzed in the database PLSDB to determine their similarity with previously isolated plasmids. There was an identity of 0.96 with plasmid NZ_CP022137.1, which was previously isolated from *Salmonella enterica* subsp. *diarizonae* serovar 65:c:z. In the case of the third contig of Canu, with a length of 75,591 bp, there was not any similarities with previously isolated plasmid. After aligning this fragment against the rest of the assemblies, it could be observed that this contig would form part of the bacterial chromosome.

For *S*. Senftenberg, one contig was obtained with Unicycler and Raven, three contigs with Flye, and five contigs with Canu. As in the case of *S*. Grancanaria, it is possible to observe a circularization of the largest contig in bandage image in the three assemblers (Figure 1). When using Flye, the second contig has a length of 6,417 bp and a 0.99 identity with 3,223 bp length plasmid NZ_CP011637.1. The results described herein are similar to those found in monophasic *S*. Typhimurium Lhica MST1 with gene duplications in Flye assembly. In the case of the third contig in Flye assembly, it presented a length of 5,352 bases before polishing and an identity of 0.99 with the 5,410 bases length plasmid NZ_CP011613.1 isolated from *Klebsiella oxytoca*. The problem of sequence duplication was not observed for this plasmid. Finally, in the case of Canu, there are two contigs of about 10,000 bases, another of about 6,000 bases, and a third of about 2,500 bases. Both contigs of 10,000 bases had an identity of 0.99 with NZ_CP011637.1, but the length of the contigs obtained with this assembler doubled the reference plasmid. There are some gene repetitions in those assemblies that were responsible for the length difference. In the other two contigs there were also gene repetitions that could indicate some problems in the assembly.

Previous studies have evaluated the effectiveness of Nanopore sequencing and *de novo* assembly for plasmid recovery (Wick and Holt, 2019; Wick et al., 2021). Wick et al. (2021) observed that Rapid library preparations (the kit used in this work) were better than ligation-based protocols for the recovery of small plasmids. In other works from Wick and Holt (2019) and Buttler and Drown (2023), different prokaryotic *de novo* assemblers were evaluated. The results showed that Raven performed poorly when recovering small plasmids. In this work, Raven was the only assembler able to recover the plasmid of monophasic *S*. Typhimurium without repetitions. However, in the case of *S*. Senftenberg, no plasmids were recovered with Raven, while two were recovered with Flye. Wick and Holt (2019) defined Flye v2.8 as “reliable, robust, and good with plasmids.” Although Flye was also good at recovering plasmids in the present work, it is important to mention that longer sequences with gene repetitions were observed. Therefore, plasmid assembly should be carefully revised when Flye was the assembler of choice.

Boostrom et al. (2022) observed that assemblies obtained with Canu presented genome sizes larger and more fragmentated than expected. Similar results were observed in this work, especially with *S*. Grancanaria and *S*. Senftenberg. In the mentioned study, the authors also observed that Canu assemblies had a higher number of single nucleotide variants, insertions, and deletions than other assemblers like Flye and/or Raven. Part of the errors in the assembly consensus sequences can be eliminated by performing assembly polishing. A previous study evaluated different polishing tools for nanopore assemblies, and the best results were obtained with Homopolish, PEPPER, and Medaka (Lee et al., 2021). Medaka was the only tool of those mentioned available in usegalaxy.eu. The other two had to be used in a Linux environment. For that reason, Medaka was used in the present work for polishing.

**Figure 2:**
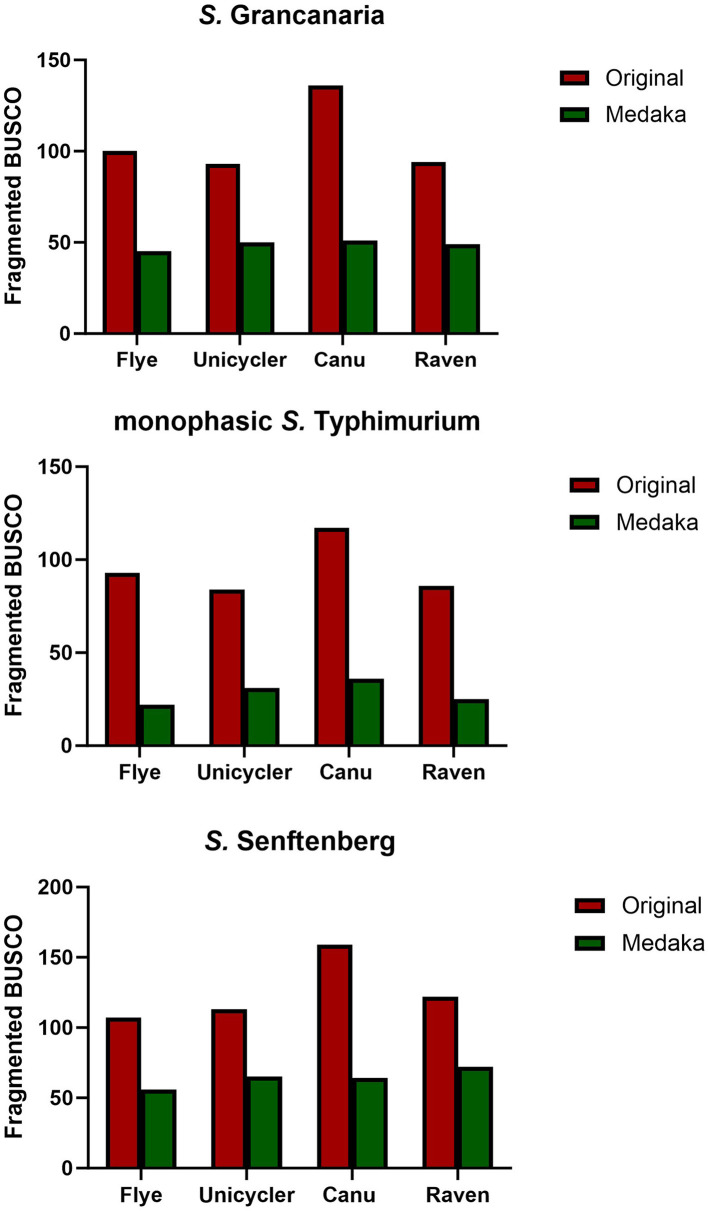
Number of fragmented BUSCO found in the different assemblies and with one and two rounds of medaka polishing.

Busco tool (Simão et al., 2015) was used for quantitative assessment of genome assembly and annotation completeness before and after polishing with Medaka. There were differences between the different assemblers. For all three strains, the original assembly obtained with Canu showed the highest number of fragmented genes (Figure 2). Medaka polishing significantly reduced the number of fragmented genes in the three strains. Canu continued to have the highest number of fragmented genes after polishing, except in the case of *S*. Senftenberg, which had the highest number of fragmented genes in the Raven assembly. It is also important to mention that the lowest number of fragmented genes was obtained when using Flye, there were consistent results with the three strains, and there were also slight differences between strains with the other three assemblers (Figure 2). The lowest number of fragmented genes was observed in monophasic *S*. Typhimurium.

### 3.3 Genome annotation

Contigs were annotated using the tool Prokka (Table 3). There were huge differences in the number of CDS and genes between the different assemblers tested in the present work. Original Canu assemblies showed the highest number of CDS and genes in the three strains. Polishing the assemblies with Medaka had a direct effect on annotation statistics. The number of CDS and genes decreased by more than one thousand after Medaka polishing. But, after polishing, Canu assemblies still had the highest number of CDS and genes. The only exception was the Unicycler assembly of monophasic *S*. Typhimurium. As previously mentioned, the assembler had some difficulties when assembling the plasmid present in this strain, and there were 107 contigs that represented the same plasmid. Therefore, there were artificial gene repetitions and this was the cause of the higher number of CDS identified in the assembly obtained with Unicycler. All the original and polished assemblies of the strains presented 22 rRNA genes as well as one tmRNA. Likewise, all the assemblies of *S*. Grancanaria had 88 tRNA. However, the other two strains showed slight differences in the number of tRNA, ranging from 87–89 in the case of monophasic *S*. Typhimurium to 85–88 in the case of *S*. Senftenberg. All the assemblies had more than 5,400 genes. This number is much higher than the genes commonly present in *Salmonella* genomes. For example, the reference genome of *S*. Typhimurium LT2 (GCF_000006945.2) presented a length of 4,951,383 bp, and 4,678 genes, a thousand less than those obtained in the annotation with Prokka. But the number of rRNA, 22, was the same in the reference and in all the assemblies of all the strains.

**Table 3:** Genome annotation of *Salmonella* isolates using the Prokka program.

	**Flye**		**Unicycler**		**Canu**		**Raven**	
	**Original**	**Medaka**	**Original**	**Medaka**	**Original**	**Medaka**	**Original**	**Medaka**
***Salmonella*** **Grancanaria**								
**CDS**	6,975	5,596	6,927	5,594	8,649	5,968	7,101	5,670
**Gene**	7,086	5,707	7,038	5,705	8,760	6,079	7,212	5,781
**rRNA**	22	22	22	22	22	22	22	22
**Repeat region**	1	2	2	2	2	2	2	2
**tRNA**	88	88	88	88	88	88	88	88
**tmRNA**	1	1	1	1	1	1	1	1
**CRISPR Arrays**	1	2	2	2	2	2	2	2
**Monophasic** ***Salmonella*** **Typhimurium**								
**CDS**	7,026	5,382	7,590	6,080	7,879	5,422	6,830	5,366
**Gene**	7,138	5,494	7,701	6,191	7,989	5,533	6,941	5,477
**rRNA**	22	22	22	22	22	22	22	22
**Repeat region**	3	3	3	3	3	3	3	3
**tRNA**	89	89	88	88	87	88	88	88
**tmRNA**	1	1	1	1	1	1	1	1
**CRISPR Arrays**	3	3	3	3	3	3	3	3
***Salmonella*** **Senftenberg**								
**CDS**	6,816	5,616	6,961	5,637	8,373	5,886	7,019	5,677
**Gene**	6,926	5,726	7,071	5,747	8,483	5,997	7,127	5,787
**rRNA**	22	22	22	22	22	22	22	22
**Repeat region**	2	2	2	2	2	2	2	2
**tRNA**	87	87	87	87	87	88	85	87
**tmRNA**	1	1	1	1	1	1	1	1
**CRISPR arrays**	2	2	2	2	2	2	2	2

One of the main issues associated with ONT technologies has been the high error rate when compared to short reading sequencing platforms. Nanopore sequencers struggle to sequence low complexity regions. It has been observed that approximately 50% of the sequencing errors are related to the presence of homopolymers (Delahaye and Nicolas, 2021). This results in insertions, mismatches, and deletion errors. Due to these sequencing errors, apparent gene frameshifts appear that have an impact on down-stream gene calling and gene annotation (Amarasinghe et al., 2020). Although polishing reduced the number of estimated genes and pseudogenes (Lee et al., 2021), it was not enough to correct all the annotation problems. Moss et al. (2020) had similar results when recovering complete, closed bacterial genomes from microbiomes using Nanopore sequencing, Flye and Canu assemblers, and Prokka for annotation. Despite the presence of these errors, the procedure was useful for obtaining draft genomes with a high degree of completeness (Athanasopoulou et al., 2022) to perform downstream characterizations.

### 3.4 *In silico* serotyping

**Figure 3:**
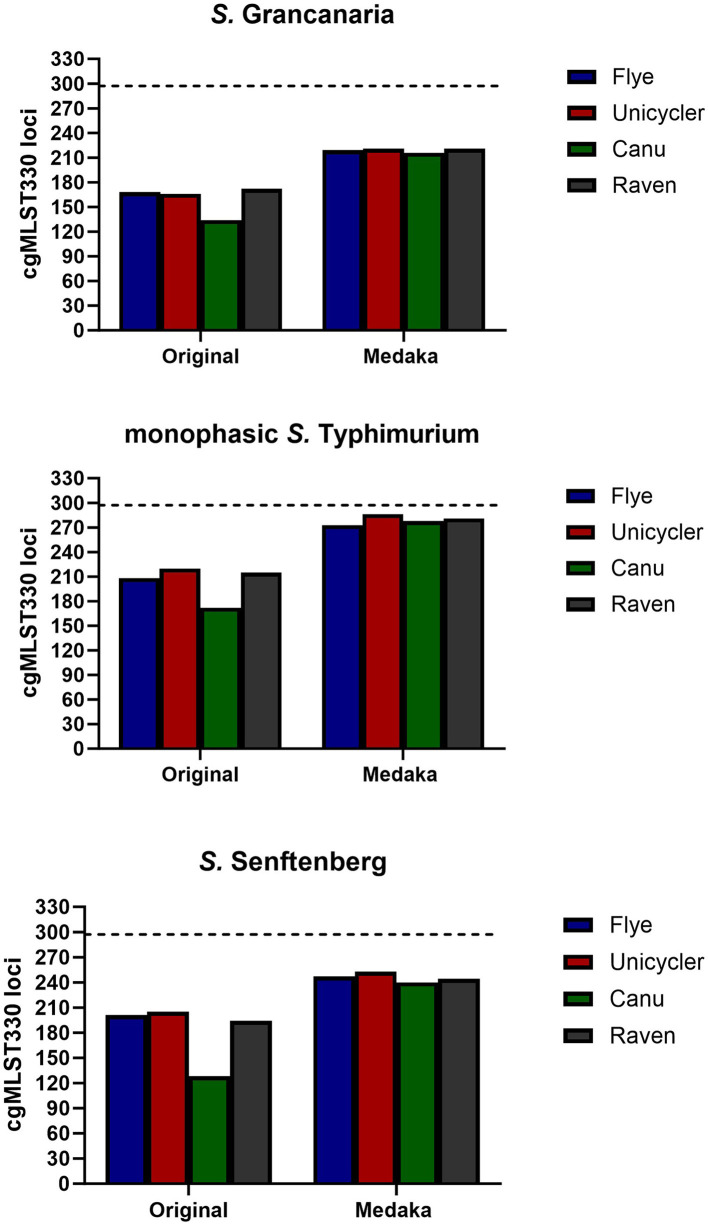
Number of cgMLST loci found with tool sistr_cmd for the different assemblies and with one and two rounds of polishing. Dotted horizontal line represents the minimum threshold for confident serovar prediction (297).

WGS is an excellent tool to perform multiple characterizations with only one wet laboratory analysis, reducing the time consumed and the reagents used. For example, with a simple bioinformatic tool *Salmonella* serotype can be determined. There are two specific tools to perform this analysis: SeqSero2 (Zhang et al., 2019), which is integrated as a web service (http://denglab.info/SeqSero2), and SISTR (Yoshida et al., 2016), available in usegalaxy. In this work, SISTR was the selected tool. There was a total correspondence between the conventional and *in silico* serotyping carried out with sistr_cmd for the three strains, with the software able to detect both somatic and flagellar antigens. This software performs serovar prediction from genome assemblies through determination of antigen genes and core genome MLST (cgMLST) using BLAST. For cgMLST, the program determines the alleles of 330 core genes selected by SISTR developers. As default in the software, the minimum threshold for confident serovar prediction is set at 297. None of the assemblies reached that threshold, probably due to mismatches related to sequencing errors (Figure 3). Out of all the assemblies, the one performed with Canu had the lowest number of alleles identified for genome serotyping. However, assembly polishing increased the number of matching alleles in all the assemblies, being particularly evident in the case of Canu. After that step, all assemblies presented a similar number of loci.

Although none of them reached the minimum threshold, in the case of monophasic *S*. Typhimurium using Unicycler and Medaka, it was possible to obtain 286 loci. Unfortunately, with the other strains, the number of loci found were lower than with monophasic *S*. Typhimurium, being around 220 and 255 loci for *S*. Grancanaria and *S*. Senftenberg respectively.

Recent studies have also evaluated the use of ONT technology for *in silico* serotyping of *Salmonella* using the GrindION and the 512 nanopore channels flow cells (Wu et al., 2021, 2023). In these studies, sequencing libraries were prepared with the Rapid Barcoding kit, the multiplex version of the kit used in the present study, and the same type of flow cell chemistry (R9.4.1). Wu et al. (2023) validated the methodology in 69 different serotypes, including serotypes with closely related antigenic formulae, representing 14 *Salmonella* groups, and found that 5 h and 30 × *Salmonella* coverage was needed for accurate serotype identification. Both tools, SeqSero2 and SISTR, were evaluated and 100% of the serotypes were correctly assigned with SeqSero2 and 98.6% with SISTR. A comparison in the number of loci found in SISTR cannot be performed between that work and the present one as Wu et al. did not indicate that information. The tool SeqSero2 can also be used with raw reads, avoiding the assembling step, unlike SISTR which only uses assembled contigs as input. However, the use of raw reads to determine the serotype when multiplexing can present some troubles due to cross-assigned reads. This results in uncorrected antigen determination as observed by Wu et al. (2021, 2023). Therefore, to avoid incorrect *in silico* serotyping when multiplexing is performed, it is better to use assembled contigs. Also, the use of single-use Flongle flow cells for sequencing, as in the present study, avoids cross-assigned reads and raw reads can be used with higher confidence. The raw reads were also used in the present study to determine the serotype with SeqSero2 with K-mer as algorithm for analysis. Monophasic *S*. Typhimurium was correctly assigned but *S*. Senftenberg was determined to be *S*. Gateshead. The software identified 9,46 O antigens instead of 1,3,19, which are the somatic antigens of *S*. Senftenberg. In the case of *S*. Grancanaria it was not possible to identify the O antigens and therefore the serotype was not determined. However, when assemblies were used (Flye + Medaka was selected), all the serotypes were correctly identified.

### 3.5 Antimicrobial resistance and virulence genes

ONT technology also represents an important opportunity to detect antimicrobial resistances. Some works have evaluated the potential of this technology in human and animal clinical diagnosis by evaluating microbial community and antimicrobial resistance profiles with promising results (Whittle et al., 2022; Ahmadi et al., 2023; Nakamura and Komatsu, 2023). The presence of resistance genes was determined using the tool ABRicate. There were differences in the resistance profiles between monophasic *S*. Thypimurium and the other two strains. *S*. Grancanaria and *S*. Senftenberg (Table 4) only carry the resistance gene *aac(6*′*)-Iaa*, which is a cryptic gene in the *Salmonella* chromosome. Apart from this gene, monophasic *S*. Typhimurium carries another six resistance genes that confer resistance to β-lactams, aminoglycosides, and sulfonamides.

**Table 4:** Resistance genes found with ABRicate tool and Resfinder database and phenotypic resistance profile.

**Strain**	**Resistance genes**	**WGS-predicted phenotype**	**Phenotypic resistance profile**
*S*. Grancanaria	*aac(6′)-Iaa*	Amikacin, tobramycin.	-
Monophasic *S*. Typhimurium	*aph(3′)-Ia, strA, aac(6′)-Iaa, strB, sul2, blaTEM-1B, tet(B)*	Amikacin, tobramycin, ampicillin, sulfamethoxazole, tetracycline, cephalothin, kanamycin, piperacillin, doxycycline, ticarcillin, minocycline, streptomycin	Ampicillin, amoxicillin clavulanic acid, ticarcillin, piperacillin, gentamicin, streptomycin, tetraciclin, doxycycline and colistin
*S*. Senftenberg	*aac(6′)-Iaa*	Amikacin, tobramycin.	-

Monophasic *S*. Typhimurium LHICA MST1 presented multiple resistance genes and therefore was selected to evaluate the effect of assembler and polishing in the detection of resistance genes using ABRicate. There were differences between original and polished assemblies (Table 5) and between different assemblers. After Medaka polishing, the percentage of similarities with reference sequences increased. The best results were obtained with Flye and Medaka polishing. There was an 100% identity with reference sequences of resistance genes. In the case of *aph(3”)-Ib_5*, 100% was possible only with polished Flye assembly. There were some differences between the ResFinder predicted phenotype and the phenotype observed. Although the predicted phenotype indicated that the strain was resistant to amikacin, tobramycin, cephalothin, and kanamycin, the phenotypic assay showed that the strain was sensitive to those antibiotics. Another discrepancy was the phenotypic resistance to colistin which had no genomic correspondence. An evaluation of the ResFinder 4.0 tool showed a Genotype-phenotype concordance ≥95%. When genotype-phenotype concordance was < 95%, it was linked to criteria for interpretation of phenotypic tests or suboptimal sequence quality (Bortolaia et al., 2020). Wu et al. (2023) found that ABRicate generated similar antimicrobial resistance genes profiles with ONT and Illumina reads for *Salmonella* strains, with optimal results when 30 × coverage, or higher, was reached. The results presented here showed that assembling with Flye along with Medaka polishing allows to identify, with 100% of identity, the antimicrobial resistance genes, demonstrating the usefulness of the proposed workflow to perform rapid antimicrobial resistance identification. Although the use of genomics for the detection and surveillance of antimicrobial resistance lags behind other applications, such as phylogenetic analysis and strain typing, the rapid advance of WGS and the cost reduction will increase the use of this methodology in routine analysis (Sherry et al., 2023). Furthermore, the quick and simple library preparation and sequencing proposed here can be very useful to accelerate the application of this technology.

**Table 5:** Percentage of identity of resistance genes found with ABRicate and Resfinder database with reference sequences (Accession column).

	**Flye**		**Unicycler**		**Canu**		**Raven**		
	**Original**	**Medaka**	**Original**	**Medaka**	**Original**	**Medaka**	**Original**	**Medaka**	**Accession**
**blaTEM-1B_1**	99.77	100	100	100	99.77	100	100	100	AY458016
**aph(6)-Id_1**	100	100	100	100	100	100	100	100	M28829
**aph(3”)-Ib_5**	99.88	100	99.88	99.88	99.75	99.88	99.88	99.88	AF321551
**sul2_3**	99.75	100	99.75	100	99.75	100	99.75	100	HQ840942
**tet(B)_2**	99.92	100	99.92	100	99.92	100	100	100	AF326777
**aac(6** **′** **)-Iaa_1**	100	100	100	100	100	100	100	100	NC_003197
**aph(3** **′** **)-Ia_9**	99.88	100	99.88	99.88	99.88	100	100	100	EU722351

ABRicate with VFDB database was used to evaluate the presence of virulence genes in *Salmonella* assemblies. There were differences between the three strains in the number of virulence factors detected (Supplementary material 1). A total of 108 virulence genes were detected in monophasic *S*. Typhimurium, 102 in *S*. Senftenberg, and 94 in *S*. Grancanaria. The same number of genes were obtained for the same strain with the four assemblers, except in the case of Canu in *S*. Senftenberg where *fep*G was not detected before polishing. The output file obtained from ABRicate provides the list of virulence genes found, the coverage, the percentage of identity, and information on the product encoded by the gene as well as if it is located in a known *Salmonella* Pathogenicity Island. As observed with resistance genes, assembly polishing reduced the number of gaps and increased the percentage identity with reference sequences. This tool is useful to get a quick idea of the virulence of the sequenced strains. In the present manuscript, monophasic *S*. Typhimurium, isolated from an outbreak in a dairy farm, presented the highest number of virulence genes. On the other hand, *S*. Grancanaria that is not related to outbreaks presents the lowest number of virulence genes.

### 3.6 Final workflow for *Salmonella* isolate sequencing

**Figure 4:**
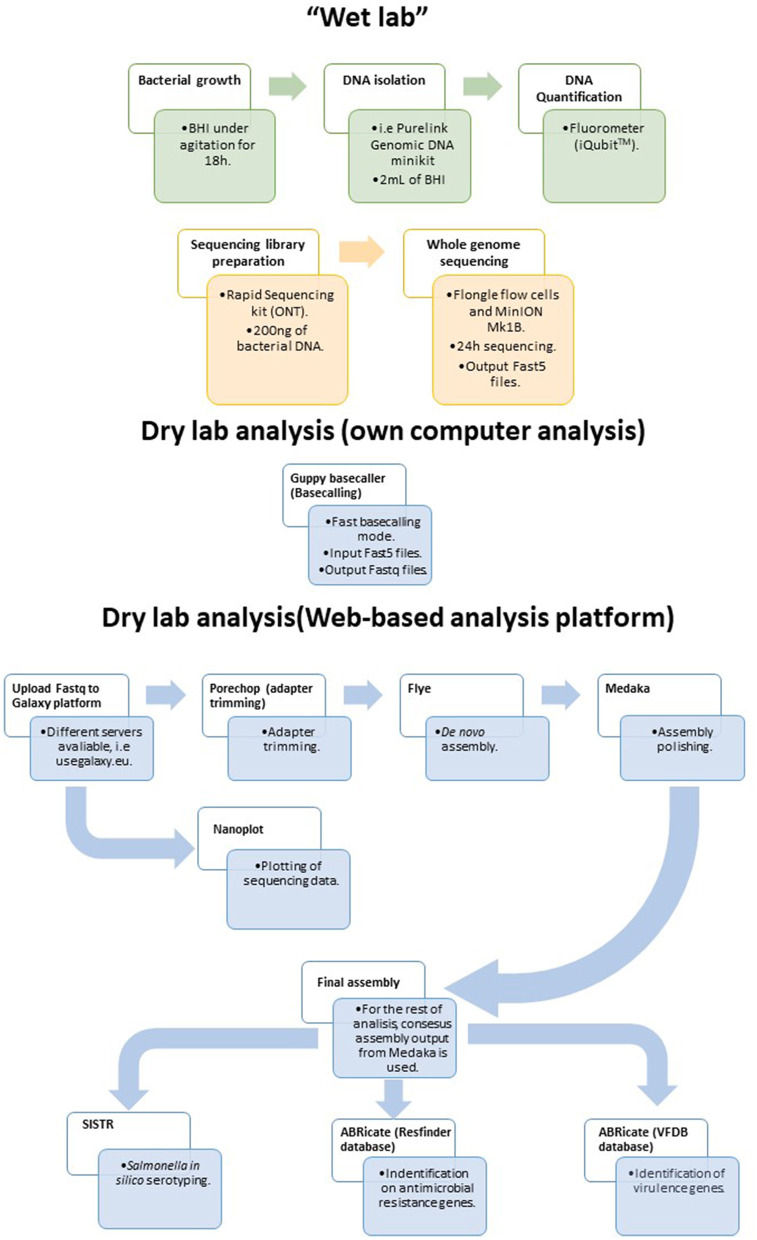
Proposed workflow for sequencing and analyzing *Salmonella* strains isolated from the food chain using the MinION and Flongle technology in combination with the web-based Galaxy platform.

Considering the results presented above, a simple and rapid protocol for WGS of *Salmonella* isolates in food laboratories is proposed herein (Figure 4). The protocol was designed to be used with generic laboratory material and by researchers/technicians with limited bioinformatic skills. For that purpose, all the bioinformatic analyses were performed in the user friendly web-based platform Galaxy. The workflow is accessible for everyone at https://usegalaxy.eu/u/alexandre_lamas/w/salmonella-assembly-and-analysis-workflow-with-ont-data by using this link, the user only has to upload the data to the Galaxy platform, select this workflow and the fastq with reads to be analyzed, and click on Run Workflow for all the analyses to be performed consecutively.

There are different kits available from ONT for WGS such as the Ligation Sequencing DNA, Ultra-Long DNA Sequencing Kit, or Rapid Sequencing kit. The latter kit is recommended for the present protocol for two main reasons. Firstly, the library preparation time is around 10 min. Ligation Sequencing DNA kit requires 60 min and Ultra-Long sequencing kit requires 200 min plus an overnight elution. The second reason is the third party materials need for each kit. While Rapid Sequencing kit requires only common laboratory materials, the ligation-based sequencing kit requires specific reagents that substantially increase sequencing price. As mentioned above, it is important not to oversee the relevance of the DNA isolation method selected, avoiding those which have steps that may increase DNA fragmentation. The equipment used is this protocol was the MinION Mk1B, which needs to be connected to a computer for sequencing runs. The advantage of this equipment is the low initial investment, at < $1,000. One of the bottlenecks in this workflow is basecalling, the transformation of raw data obtained during sequencing in nucleotides. The Guppy basecaller developed by ONT can perform basecalling in two modes, CPU and GPU. In Windows, users can also use the CPU mode, which is hundreds of times slower than GPU mode, which can be used in a Linux environment. ONT also offers another portable MinION, the model Mk1C with a fully integrated computer and screen, which eliminates the need for a computer to generate and analyze sequencing data. This is a user-friendly model that can perform basecalling in GPU mode, reducing the analysis time. But the initial investment for this equipment is five times higher than that of the other model. Regardless of which model is used, the same bioinformatic workflow can be followed.

Focusing on the flow cells, this protocol was based on the usage of Flongle flow cells. In the case of bacterial genomes of *Salmonella* size (5 Mb), a single Flongle run is enough to obtain sufficient coverage. In addition to this, more than one strain can be sequenced simultaneously but there is a risk of not achieving the required coverage. The theorical output under optimal conditions for these flow cells was reported to be up to 2.8 Gb, however, in our hands the highest output obtained was 0.49 Gb (bases after basecalling). Therefore, running one strain at a time is recommended. Multiplexing may be suitable for foodborne pathogens with smaller genome sizes like *L. monocytogenes*. The final workflow consists of Guppy basecalling, followed by Flye assembly and one round of Medaka polishing. This decision was based on different reasons. First, the Flye assembler recovered small plasmids that were not recovered by the other assemblers, it was the fastest assembler of all those used, and also showed the best performance in previous studies (Wick and Holt, 2019; Wick et al., 2021; Buttler and Drown, 2023). It is important not to oversee the fact that in some cases there were gene duplication problems. Additionally, Flye with Medaka was the only combination that found an 100% percentage of identity between resistance genes present in monophasic *S*. Typhimurium and reference genes. Finally, ONT bacterial genome assembly workflow (ONT, 2022) recommend Flye assembly with one round of Medaka polishing. This assembly showed good results for serotyping and genomic antimicrobial resistance and virulence profiling. Therefore, by using a Flongle flow cell, which has a list price of $90, and a Rapid Sequencing kit, it is possible to perform WGS of *Salmonella* strains isolated from the food chain in a single wet lab analysis. Then, with a few clicks, it is possible to fully characterize the strains.

Finally despite all the benefits of this technology, we are also aware of its limitations. The presence of systematic errors, especially in homopolymer regions, makes the annotation of the genome difficult and may cause problems with some genomic characterization tools. In the present protocol, fast basecalling using Guppy basecaller was used. This mode is the least accurate of those available but also the fastest. This is especially relevant when using CPU mode on a laptop computer to perform basecalling. The use of a high-accuracy or super-accurate model, with the number of reads obtained in this work, can extend this step for days, limiting the fast response time. In case the researchers want to perform a deeper genomic analysis, they should choose the more accurate methods. It is also important to mention that ONT is continuously improving both chemistry and analysis software that minimize these errors and bring its accuracy closer to short-reading sequencing. Now ONT has introduced the Q20+ chemistry (with new kits and flow cells) that outperforms the chemistry types and gives better results than that obtained here with the older chemistry. Also, ONT is replacing Guppy basecaller with Dorado basecaller, which provides better results. Due to the continuous innovation carried out by ONT, it is always necessary for the researcher to be aware of the new improvements introduced.

## 4 Conclusions

The combination of Flongle flow cells and MinION Mk1B with Rapid Sequencing kit can be successfully used for WGS sequencing of foodborne pathogens such as *Salmonella* spp. at a lower price. In addition, the use of the community-driven, web-based analysis platform Galaxy for bioinformatic analysis allows laboratories with no bioinformatic skills to implement this technology. The *de novo* assembly, *in silico* serotyping, and antimicrobial and virulence gene detection can be performed with a single click. The workflow developed herein is a cost-effective, powerful tool to be implemented in the food production chain by food safety laboratories or even in the food industry to control and characterize *Salmonella* isolates.

## Data availability statement

The datasets presented in this study can be found in online repositories. The names of the repository/repositories and accession number(s) can be found below: https://www.ncbi.nlm.nih.gov/, PRJNA990827.

## Author contributions

AL: Conceptualization, Formal analysis, Investigation, Methodology, Writing – original draft. AG-M: Writing – original draft. AP: Investigation, Writing – review & editing. AC: Writing – review & editing. CF: Conceptualization, Formal analysis, Writing – review & editing.
